# Application of Next-Generation Sequencing for the Genomic Characterization of Patients with Smoldering Myeloma

**DOI:** 10.3390/cancers12051332

**Published:** 2020-05-23

**Authors:** Martina Manzoni, Valentina Marchica, Paola Storti, Bachisio Ziccheddu, Gabriella Sammarelli, Giannalisa Todaro, Francesca Pelizzoni, Simone Salerio, Laura Notarfranchi, Alessandra Pompa, Luca Baldini, Niccolò Bolli, Antonino Neri, Nicola Giuliani, Marta Lionetti

**Affiliations:** 1 Department of Oncology and Hemato-oncology, University of Milan, 20122 Milan, Italy; martina.manzoni88@gmail.com (M.M.); luca.baldini@unimi.it (L.B.); niccolo.bolli@unimi.it (N.B.); marta.lionetti@unimi.it (M.L.); 2 Department of Medicine and Surgery, University of Parma, 43126 Parma, Italy; valentina.marchica@unipr.it (V.M.); paola.storti@unipr.it (P.S.); l.notarfranchi@gmail.com (L.N.); nicola.giuliani@unipr.it (N.G.); 3 Department of Clinical Oncology and Hematology, Fondazione IRCCS Istituto Nazionale dei Tumori, 20133 Milan, Italy; bachisio.zic@gmail.com; 4 Hematology, “Azienda Ospedaliero-Universitaria di Parma”, 43126 Parma, Italy; gsammarelli@ao.pr.it (G.S.); giannalisat@gmail.com (G.T.); 5 Hematology Unit, Fondazione IRCCS Ca’ Granda Ospedale Maggiore Policlinico, 20122 Milan, Italy; francesca.pelizzoni1@studenti.unimi.it (F.P.); simone.salerio@gmail.com (S.S.); alessandra.pompa@policlinico.mi.it (A.P.)

**Keywords:** multiple myeloma, premalignant stages, next-generation sequencing

## Abstract

Genomic analysis could contribute to a better understanding of the biological determinants of the evolution of multiple myeloma (MM) precursor disease and an improved definition of high-risk patients. To assess the feasibility and value of next-generation sequencing approaches in an asymptomatic setting, we performed a targeted gene mutation analysis and a genome-wide assessment of copy number alterations (CNAs) by ultra-low-pass whole genome sequencing (ULP-WGS) in six patients with monoclonal gammopathy of undetermined significance and 25 patients with smoldering MM (SMM). Our comprehensive genomic characterization highlighted heterogeneous but substantial values of the tumor fraction, especially in SMM; a rather high degree of genomic complexity, in terms of both mutations and CNAs, and inter-patient variability; a higher incidence of gene mutations and CNAs in SMM, confirming ongoing evolution; intraclonal heterogeneity; and instances of convergent evolution. ULP-WGS of these patients proved effective in revealing the marked genome-wide level of their CNAs, most of which are not routinely investigated. Finally, the analysis of our small SMM cohort suggested that chr(8p) deletions, the DNA tumor fraction, and the number of alterations may have clinical relevance in the progression to overt MM. Although validation in larger series is mandatory, these findings highlight the promising impact of genomic approaches in the clinical management of SMM.

## 1. Introduction

The recent progress in next-generation sequencing (NGS) techniques applied to multiple myeloma (MM) has prompted comprehensive characterizations of genomic alterations in malignant plasma cells (PCs) through clinical-grade approaches [1,2]. Different types of genomic lesions may contribute to myelomagenesis and have a prognostic impact. Their detection, however, currently requires the use of multiple molecular techniques (fluorescence in situ hybridization (FISH), Sanger sequencing, and single nucleotide polymorphism arrays), which makes analysis expensive and technically demanding. Furthermore, a large number of PCs is required. This may represent a limitation, especially in the pre-malignant stages of the disease, i.e., monoclonal gammopathy of undetermined significance (MGUS) and smoldering MM (SMM). MGUS and SMM, in fact, are characterized by a lower tumor burden than overt MM. Therefore, a poor recovery of clonal CD138^+^ cells from the bone marrow (BM) biopsy may preclude a thorough genomic characterization of these patients. On the contrary, genomic analysis is proving increasingly important for better understanding the biological determinants of the evolution of MM precursor clinical entities [3,4,5] and this could translate into a more effective definition of patients at high-risk for progression, thus maximizing the benefit of early treatment [6,7]. While whole-genome sequencing approaches cannot be employed in routine clinical care due to their cost and turnaround time, several groups have shown the accuracy of custom target NGS solutions for a streamlined detection of copy number abnormalities (CNAs), translocations at immunoglobulin heavy chain (IGH) locus, and gene mutations in MM [8,9,10,11,12]. These approaches have mainly been evaluated for patients with overt MM. Here, we present an extensive NGS-based genomic characterization of asymptomatic form cases, i.e., six MGUS and 25 SMM patients, assessing its accuracy compared to gold standard diagnostic techniques and its prognostic value compared to the risk stratification criteria currently based on laboratory values.

## 2. Results

### 2.1. Tumor Load and Tumor Mutational Burden

**Figure 1:**
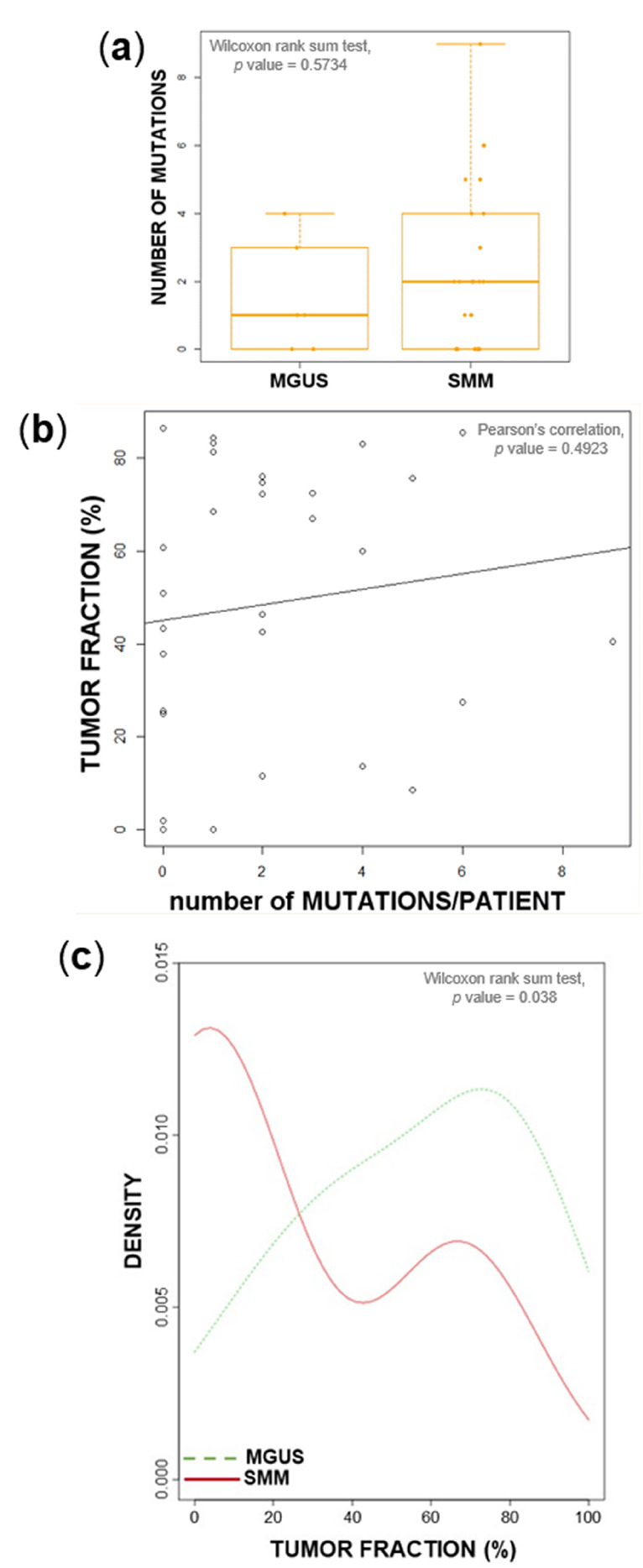
Relationship between the tumor fraction, mutation burden, and disease stage. (**a**) Number of detected mutations in six monoclonal gammopathy of undetermined significance (MGUS) and 25 smoldering multiple myeloma (SMM) patients. (**b**) The number of called mutations in genomic DNA from CD138^+^ bone marrow (BM) plasma cells of each patient did not correlate with the estimated tumor fraction. (**c**) Density plot of estimated tumor fractions in six MGUS and 25 SMM patients.

Ultra-low pass whole-genome sequencing (ULP-WGS) and ultra-deep targeted NGS of a 56-gene panel were performed at an average coverage across all samples of 0.1× and 3062×, respectively. Our targeted panel identified a median of one mutation per patient in MGUS (range: 0–4) and two in SMM (range: 0–9), without a statistically significant difference between the distributions of the two groups (Figure 1a). Approximately one third of patients were devoid of mutations in the sequenced genes, with similar proportions in MGUS and SMM. In asymptomatic cases, the detection of somatic gene mutations in BM CD138^+^ cells could be undermined by the presence of normal PCs in the sample. To address this issue, we estimated the tumor fraction by ichorCNA analysis of ULP-WGS data for each patient, and correlated it to the number of variants identified. No correlation was identified (Figure 1b), and many patients did not carry any mutation, despite a high tumor fraction (over 25% in 7/10 non-mutated cases). This suggests that, at this depth of sequencing, the test is sensitive enough to account for contamination of the sample and return accurate results. On the other hand, the occurrence of mutations with considerable allelic frequencies in patients with very low estimated tumor fractions is, in all likelihood, justifiable with the fact that, since ichorCNA estimates the percentage of tumor-derived DNA based on the detection of somatic CNAs, this analytical tool may underestimate tumor fractions in patients devoid of clonal CNAs (as non-hyperdiploid, IGH-translocated asymptomatic stages are likely to be). In general, tumor fractions were distributed over a wide range of values, and were globally higher in SMM (median: 60%, range: 0–86.5%) than in MGUS (median: 8%, range: 0–68.5%) (Wilcoxon rank sum test, *p* = 0.038, Figure 1c). Tumor fractions did not significantly correlate with BM PC infiltration (Figure S1).

### 2.2. Mutational Landscape

**Figure 2:**
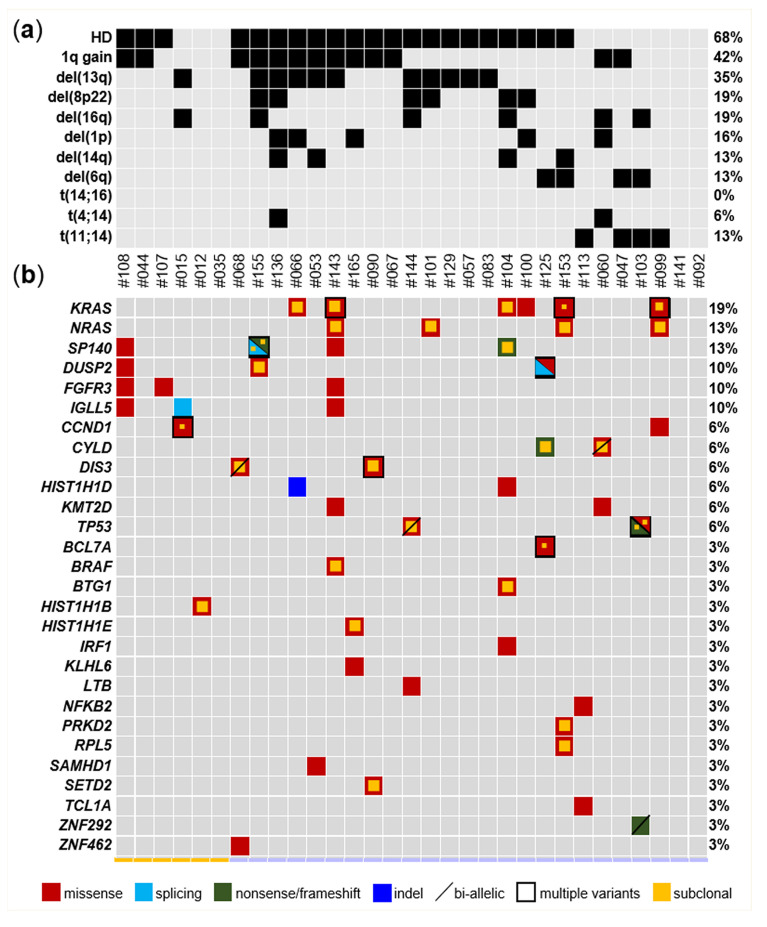
Overview of genomic aberrations and gene mutations in asymptomatic multiple myeloma. (**a**) Heatmap of selected chromosomal copy number alterations (CNAs), as assessed by ultra-low-pass whole genome sequencing (ULP-WGS), and immunoglobulin heavy chain locus (IGH) chromosomal translocations, as assessed by fluorescence in situ hybridization (FISH), in the six MGUS and 25 SMM patients. Only CNAs occurring in ≥4 samples are plotted; chromosome arms within which the CNAs (with variable extensions) localize are indicated. Gray squares indicate an absence of alterations, and black ones indicate their occurrence. (**b**) Mutated genes, color coded for missense (red), splice-site (light blue), nonsense/frameshift (green), indel (orange). A diagonal bar highlights mutations occurring in a gene with a copy number of 1. In the case of multiple variants detected, the squares are countered in black. A smaller internal yellow square denotes subclonal mutations. Only genes mutated in at least one sample are plotted. Each column represents one tumor sample and each row represents one chromosomal alteration/gene. MGUS samples are indicated in yellow, and SMM samples in lilac. The percentage of tumors carrying each alteration is provided on the right.

Our targeted NGS approach identified mutations in 68% of patients (21/31) and in half of the genes included in the design (Figure 2; Table S1). Positive patients more often carried multiple variants (chi-square test, *p* = 0.012) involving different genes; the most extensively mutated patient was ID#143, for whom we identified nine variants in seven genes. In terms of the mutation type, variants were mostly missense variants. The *KRAS* gene was mutated in 19.4% of patients (6/31); *NRAS* and *SP140* in 12.9% (4/31 each); and *DUSP2*, *FGFR3*, and *IGLL5* in 9.7% (3/31 each). Out of the 66 mutations, 31 were clonal and 35 subclonal (so defined if involving ≥90% or <90% of the tumor fraction estimated by ULP-WGS, respectively). Notably, mutated samples more often carried co-occurring clonal and subclonal variants, even within the same gene. Of note, *KRAS* was targeted by multiple mutations in three patients. In particular, we found clonal Q61H and subclonal K117N and Q22K in ID#99; G12C, G12D, and Y64D, all at subclonal levels, in ID#143; and clonal G13D and Q61H and subclonal G12V in ID#153. Interestingly, each of these three patients carried one or more additional mutated genes belonging to the mitogen-activated protein kinase (MAPK) pathway, i.e., *NRAS* (subclonal Q61R in ID#99, Q61K in ID#143, and Q61L in ID#153) and *BRAF* (subclonal V600E in ID#143) (Figure S2). Altogether, these data support a complex and spontaneously evolving subclonal structure of MM, even in an asymptomatic setting, with instances of convergent evolution and high-risk lesions of prognostic value in case treatment is initiated.

### 2.3. Chromosomal Alterations

**Figure 3:**
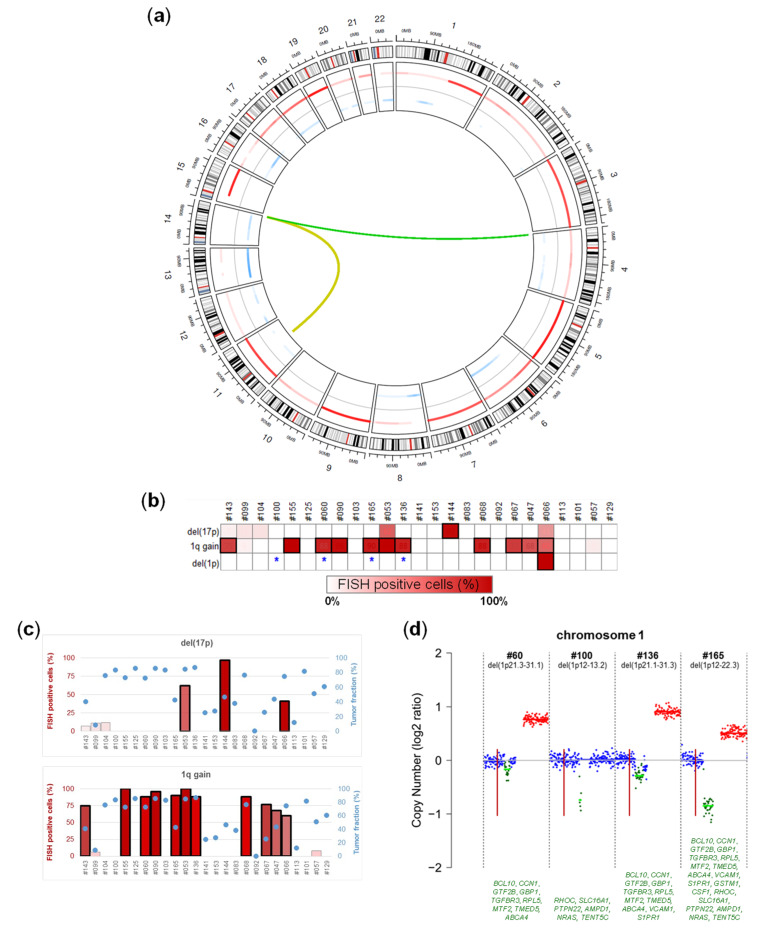
Chromosomal alterations characterizing the genome of asymptomatic multiple myeloma. (**a**) Circos plot showing genome-wide copy number abnormalities (from ULP-WGS) and chromosomal translocations involving the IGH locus (from FISH analysis) found in tumor samples from six MGUS and 25 SMM patients. Autosomes are arranged around the circle, starting from the top at chromosome 1 and continuing clockwise to chromosome 22. Copy number data are presented on the inside of the circle, showing gains/amplifications (first track, red), normal copy number (second track, grey), and deletions (third tack, light blue). Immunoglobulin heavy chain locus (IGH@) translocations are shown as green lines emerging from chromosome 14 to their respective partner chromosomes. The color intensity of gained and lost segments and the thickness of the lines indicating IGH translocations heighten with an increasing frequency of that anomaly in the cohort. (**b**) Heatmap representing the percentage of cells carrying each of the indicated chromosomal copy number alterations, as detected by FISH analysis. Each column represents one tumor sample and each row represents one CNA. Samples that tested positive for the corresponding lesion by ULP-WGS analysis are framed. N.a., not available. (**c**) Bar graphs representing the percentage of cells carrying each of the indicated chromosomal copy number alterations, as detected by FISH analysis (left vertical axis), in the 25 SMM patients. IchorCNA-estimated tumor fraction in the plasma cell (PC) gDNA of each patient is indicated by a light-blue circle (right vertical axis). Samples that tested positive for the corresponding lesion by ULP-WGS analysis are framed. (**d**) Chromosome 1 copy ratios computed by ichorCNA from ULP-WGS data in four patients (marked by an asterisk in **b**) tested negative for del(1p) by FISH analysis. Amplification (red), loss (green), and copy neutral (blue) are indicated. The horizontal lines in light green indicate subclonal calls. The vertical red bar marks the location of the FISH probe. The main genes mapped in the deleted region in each patient are listed in the lower part of the figure.

For a comprehensive view of the genomic alterations in our cohort, we characterized recurrent IGH translocations by FISH and genome-wide CNAs by ichorCNA analysis of ULP-WGS data. Information related to IGH translocations and selected CNAs recurrently associated with MM are visualized in the upper heatmap in Figure 2, while Figure 3a and Figure S3 depict genome-wide CNAs. Four and two SMM patients carried t(11;14) and t(4;14), respectively. Concerning CNAs, the hyperdiploid status (defined as trisomy of two out of odd chromosomes 3, 5, 7, 9, 11, 15, 19, and 21) was the most recurrent one, occurring in 68% of patients. The median number of involved odd chromosomes present in extra copies was four and five in hyperdiploid MGUS and SMM cases, respectively. The other most recursively detected lesions were 1q gain/amplification (42% of cases), del(13q) (35%), del(8p) and del(16q) (19% each), del(1p) (16%), and del(14q) and del(6q) (13% each). The incidence of these CNAs was higher in SMM than in MGUS (Wilcoxon rank sum test, *p* = 0.04). Interestingly, we found instances of bi-allelic events in *ZNF292* in ID#103, *DIS3* in ID#68, *TP53* in ID#144, and *CYLD* in ID#060, which is a finding often associated with advanced stages [13]. CNAs at 1p, 1q, and 17p, as predicted by ULP-WGS, were compared with FISH data, available for the 25 SMM patients and based on signals obtained in purified BM PCs at cytobands 1p32, 1q21, and 17p13, respectively (Figure 3b–d). We found almost full concordance in terms of gains/amplifications detected by FISH and ULP-WGS at chromosome arm 1q (Figure 3b,c), with the exception of two samples, in which the gain, harbored by 6% and 8% of interphase nuclei, respectively, was not detected by ULP-WGS. In agreement with FISH, ULP-WGS revealed a deletion of 17p13.1-pter in patient ID#144. However, it failed to detect a 17p13 monoallelic deletion found in very small subclones in three FISH-positive patients (7% of positive cells in ID#143, 11% in ID#99, and 12% in ID#104), as well as in two samples in which FISH identified 62% (ID#53) and 41% (ID#66) of positive cells, respectively. Since the percentages of FISH-positive cells were quite high, and therefore theoretically appreciable by ULP-WGS, we believe that this discrepancy in the latter two samples may depend on very focal deletions which have been reported to occur [14]. Therefore, ULP-WGS analysis by ichorCNA, which, by default, computes log_2_ copy ratios for genomic windows of 1Mb [15], may miss the deletion. To verify this hypothesis, we re-analyzed the ULP-WGS data of these two samples by ichorCNA, setting genomic windows equal to 50 kb. In this way, we managed to detect the deletion in both patients, confirming its limited extension (1.7 Mb in ID#53 and 5.9 Mb in ID#66, respectively) and the involvement of the *TP53* gene (Figure S4). Furthermore, ULP-WGS was able to provide more information than FISH concerning interstitial deletions of chromosome arm 1p (Figure 3d). Both methods detected a deletion in sample ID#66. In four additional FISH-negative patients, ichorCNA identified interstitial 1p deletions with centromeric localization compared to the probe used for FISH, and mapping at 1p21.3-p31.1 (ID#060), 1p12-p13.2 (ID#100), 1p21.2-p31.3 (ID#136), and 1p12-p22.3 (ID#165), respectively. We confirmed these 1p interstitial deletions as true events by using targeted sequencing data of the 56-gene panel (Figure S5).

### 2.4. Prognostic Value of Genomic Findings

**Figure 4:**
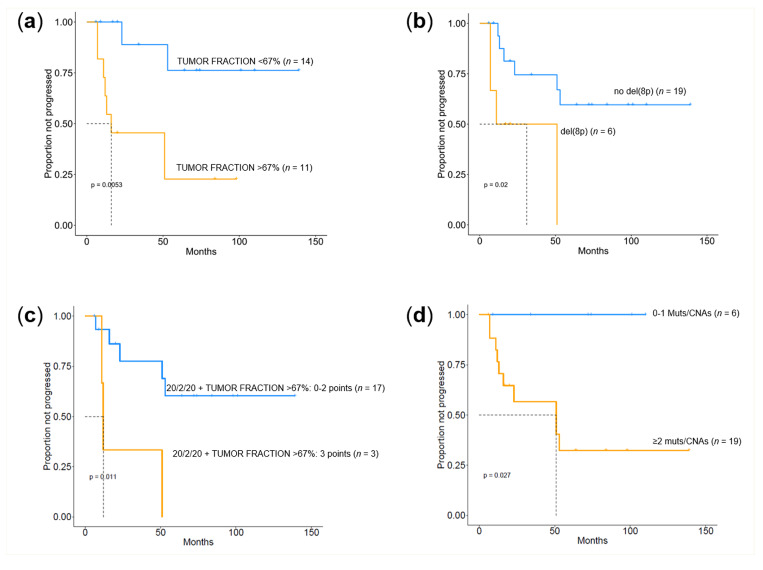
Kaplan–Meier curves of time to progression (TTP) by next-generation sequencing (NGS)-derived molecular information in SMM. Kaplan–Meier curves of TTP according to the ichorCNA-estimated tumor fraction of bone marrow (BM) plasma cell (PC) genomic DNA (**a**), the occurrence of deletions in chromosome arm 8p (**b**), the 20/2/20 model integrated with the tumor fraction of BM PC genomic DNA (**c**), and the number of mutational and CNA events carried by each patient (**d**).

We then assessed whether the information obtained from targeted NGS and ULP-WGS showed any correlation with the clinical outcome of the patients, restricting the analysis to the 25 SMM cases. Gene mutations did not correlate with time to progression: this applied to the type of gene, to the number of genes mutated, and to their allelic fraction. However, we found that patients with a tumor fraction higher than 67% (i.e., the tumor fraction value identified by an ROC analysis as the best discriminating cut-off between progressing and non-progressing cases) had a significantly shorter time to progression (TTP) (median of 16 months vs. not reached, *p* = 0.0053) (Figure 4a). Concerning CNAs, the occurrence of chromosome 8p deletion seemed to be associated with a more rapid progression to symptomatic MM (median of 11 months vs. not reached, *p* = 0.02) (Figure 4b). The percentage of BM PCs correlated with the risk of progression, as expected. However, the recently proposed risk classification of SMM stratifying patients into three groups based on the occurrence of BMPC > 20%, M-protein > 2 g/dL, and free light chain ration (FLCr) > 20 (20/2/20 model) [16], was not able to discriminate different intervals of TTP in our small series. To assess whether the information obtained from our genomic analysis might have some relevance in the context of current prognostic models based on surrogates of tumor burden, we tried to integrate the 20/2/20 model with the tumor fraction estimate. We found that by assigning an additional point to patients with a DNA tumor fraction higher than 67%, the high-risk group (score ≥ 3) had a significantly shorter TTP (median of 12 months vs. not reached, *p* = 0.011) (Figure 4c). Lastly, when we combined mutational data with genome-wide CNAs, we found that the presence of two or more events (mutations and/or CNAs) was associated with faster disease progression (median of 51 months vs. not reached, *p =* 0.027) (Figure 4d).

## 3. Discussion

Monoclonal gammopathy of undetermined significance (MGUS) and SMM represent intermediate steps in the development of overt MM. MGUS progresses at a constant and low rate of 1%/year [17]. SMM is clinically more heterogeneous, including both MGUS-like and MM-like patients at a high risk of developing a myeloma-defining event within the first 2 years of diagnosis [18]. Different clonal evolution patterns characterizing these two clinical subsets of SMM patients have been identified by recent NGS studies [5,19]. It follows that genomic analysis could contribute to a better understanding of the biological basis of the evolution of MM precursor disease, as well as a refinement of the definition of high-risk patients. To assess the feasibility and value of such studies, in this research we performed a targeted gene mutation analysis and a genome-wide CNA assessment aimed at a comprehensive view of genomic alterations in six MGUS and 25 SMM patients.

Our series of asymptomatic cases was characterized by heterogeneous but substantial values of the tumor fraction, especially in SMM. This was independent of the number of mutations identified in each sample, which ensures the reliability of our data and supports the use of NGS approaches for an in-depth genomic characterization of these patients. Overall, our analyses of asymptomatic forms revealed a rather high degree of genomic complexity, in terms of both mutational and chromosomal aberrations, and inter-patient variability. The median number of variants was one in MGUS and two in SMM patients. SMM patients presented a higher incidence of CNAs and specific aneuploidies in hyperdiploid samples, confirming the ongoing evolution in these asymptomatic stages, as reported in preliminary reports based on FISH [20]. More recent high-throughput studies showed that MGUS is less genetically complex than MM, and high-risk SMM is similar to presenting MM in terms of mutations [21] and CNAs [22]. In SMM samples, we observed a somewhat binary distribution, whereby patients either showed no mutations or multiple mutations. The mixture of clonal and subclonal events concerning both gene mutations and CNAs in the majority of patients was suggestive of intraclonal heterogeneity, confirming that this is a feature of all disease stages [5,21,23]. Interestingly, while the six MGUS patients were devoid of mutations in genes of the MAPK pathway, this was recurrently targeted in SMM cases, with *KRAS* and *NRAS* being the two top-ranking genes in terms of mutational incidence. An SMM sample also carried a *BRAF* V600E mutation. Furthermore, our in-depth analysis highlighted examples of convergent evolution that included multiple mutations within the same gene or pathway found in the same patient: among these, there were three cases harboring MAPK mutations, and one patient with two *TP53* mutations, similar to what has been reported in the symptomatic setting [24,25,26,27]. ULP-WGS has proven to be effective in highlighting how the karyotype of asymptomatic patients is altered at the genome-wide level, also detecting recurrent CNAs that are not routinely investigated by FISH. This allowed us to identify tumor suppressor genes, such as *TP53* and *CYLD*, which were frequently hit by bi-allelic events, something previously described in advanced disease. Furthermore, interstitial deletions in chr(1p) not detected by FISH could be identified by ULP-WGS, further confirming how genomic approaches can outperform FISH [4]. Although ULP-WGS showed a reduced sensitivity in the detection of highly subclonal deletions, this is likely to be of little relevance in laboratory and clinical routine practice, since the current threshold for the detection of FISH events is 10% and even much higher according to consensus criteria for chr(17p) deletion [28]. Interestingly, the more extensive information provided by the ULP-WGS approach appears to be of clinical value in our small SMM cohort. In fact, we could assign a prognostic value to chr(8p) deletions; to the tumor fraction of purified BM PCs; and, in general, to the number of genomic events, mutations, and/or CNAs, either alone or in combination with the revised IMWG SMM risk stratification. Although the limited size of the present cohort and the lack of validation in larger independent series impose caution in extending the validity of the specific clinical correlates identified, these findings highlight the promise of genomic approaches in the clinical management of SMM.

## 4. Materials and Methods

### 4.1. Patients

The study was based on a series of six patients with MGUS and 25 with SMM admitted to our institution. MGUS and SMM were diagnosed according to the International Myeloma Working Group (IMWG) revised criteria [29] and SMM patients were stratified by recently proposed risk factors for progression [16]. Patients‘ clinical and molecular characteristics are reported in Table S2. None of the patients enrolled in this study had previously received anti-MM therapy, and in all cases, sampling was done under conditions of clinically stable disease. The study was approved by the local Ethics Committee (Provision n.575 dated 29/03/2018) and written informed consent was obtained from all of the patients involved in the study. The study was conducted according to good clinical practice and the ethical principles outlined in the Declaration of Helsinki. BM mononuclear cells (MNCs) were obtained from BM aspirates, after gradient centrifugation with Ficoll solution (Lympholyte^®^ Cell Separation Media, Cedarlane, Canada). CD138^+^ PCs were isolated from BM MNCs by an immunomagnetic method with anti-CD138 monoclonal antibodies (mAbs) conjugated with microbeads (Miltenyi Biotech; Bergisch-Gladbach, Germany). For the purification of genomic DNA, we used the AllPrep DNA/RNA/miRNA isolation kit (Qiagen, Hilden, Germany), following the manufacturer’s protocol.

### 4.2. Next-Generation Sequencing

We collected tumor gDNA from CD138^+^-purified BM PCs. The targeted resequencing gene panel included coding exons and splice sites of 56 genes that emerged as drivers from a recent analysis of whole genome/exome data in more than 800 MM patients [30] (target region: 112 kb: *ACTG1, BCL7A, BHLHE41, BRAF, BTG1, CCND1, CDKN1B, CYLD, DIS3, DTX1, DUSP2, EGR1, FAM46C, FGFR3, FUBP1, HIST1H1B, HIST1H1D, HIST1H1E, HIST1H2BK, IGLL5, IRF1, IRF4, KLHL6, KMT2B, KRAS, LCE1D, LTB, MAX, NFKB2, NFKBIA, NRAS, PABPC1, PIM1, POT1, PRDM1, PRKD2, PTPN11, RASA2, RB1, RFTN1, RPL10, RPL5, RPRD1B, RPS3A, SAMHD1, SETD2, SP140, TBC1D29, TCL1A, TGDS, TP53, TRAF2, TRAF3, XBP1, ZNF462, ZNF292*). Ultra-deep NGS was performed on MiSeq (Illumina, Hayward, CA, USA) using the CAPP-seq library preparation strategy (NimbleGen, Roche, Madison, WI, USA), as previously described [31,32]. The germline function of VarScan2 *mpileup2cns* was used to identify non-synonymous somatic mutations, and a stringent bioinformatic pipeline was developed and applied to filter out sequencing errors (detection limit 3 × 10^−3^). Details of the experimental procedures are given in Supplementary Methods. For ULP-WGS, libraries were prepared using the Kapa HyperPlus kit (Roche, Madison, WI, USA) with SeqCap Library Adapters starting with 400 ng of gDNA. Up to 24 libraries were pooled and sequenced using 200 bp paired-end runs on a MiSeq (Illumina). In order to estimate the quality and presence of a tumor, we performed ULP-WGS with an average genome-wide fold coverage of 0.1×. We analyzed the depth of coverage to estimate large-scale CNAs and the fraction of tumor DNA using ichorCNA [15] with a panel of 34 normal samples.

### 4.3. Fluorescence In Situ Hybridization (FISH) Analysis

Interphase FISH analyses were performed as previously described [33] on purified BM PCs.

### 4.4. Statistical Analyses

Time to first progression (TTP) was calculated from diagnosis to the first occurrence of any myeloma-defining event (event) or to last follow-up (censoring). TTP analyses were performed using the Kaplan–Meier method. The statistical significance of associations between individual variables and TTP was calculated using the log-rank test.

## 5. Conclusions

Overall, our findings demonstrate that, starting from a limited amount (400 ng) of DNA from purified BM PCs of asymptomatic patients, a highly informative genomic analysis is feasible. To entirely replace FISH, in the future, more sophisticated targeted designs could be conceived, including chromosomal regions of recurrent CNAs and the IGH locus, to enable the detection of IGH translocations. Preliminary studies have proven promising in this setting [8,9,10,11,12]. Alternatively, as sequencing costs will decrease, CNAs and IGH translocations could be detected by a shallow, long-insert WGS. This would also produce data on the tumor fraction, which proved to be prognostically relevant in our series. Although the presented data need validation in an independent cohort, the finding that the tumor fraction did not correlate with the percentage of plasma cell infiltration in the BM makes its prognostic value even greater, and suggests that it may be worthwhile to consider it to improve current SMM risk stratification.

## Supplementary Materials

The following are available online at https://www.mdpi.com/2072-6694/12/5/1332/s1: Supplementary Methods; Figure S1: Tumor fraction and BM PC infiltration; Figure S2: MAPK gene mutations; Figure S3: Heatmap of altered DNA regions in six MGUS and 25 SMM samples, as assessed by means of ichorCNA analysis of ULP-WGS data; Figure S4: Detection of *TP53* gene deletion by ichorCNA analysis of ULP-WGS data; Figure S5: Detection of 1p-deletions based on targeted NGS data; Table S1: Non-synonymous mutations discovered by targeted gene mutation analysis; Table S2: Patients’ characteristics.
