# Supplementary material for: Application of Next-Generation Sequencing for the Genomic Characterization of Patients with Smoldering Myeloma
Source: Cancers (Basel). 2020 May 23;12(5):1332. doi: 10.3390/cancers12051332 (PMC7281620; doi:10.3390/cancers12051332)
Supplement: Supplementary file 1 [file cancers-12-01332-s001.pdf]

cancers

MDPI

*Article*

# Application of Next-Generation Sequencing for the Genomic Characterization of Patients with Smoldering Myeloma

**Martina Manzoni, Valentina Marchica, Paola Storti, Bachisio Ziccheddu, Gabriella Sammarelli, Giannalisa Todaro, Francesca Pelizzoni, Simone Salerio, Laura Notarfranchi, Alessandra Pompa, Luca Baldini, Niccolò Bolli, Antonino Neri, Nicola Giuliani and Marta Lionetti**

## Supplementary Materials

## Supplementary Methods

### *Library Design, CAPP-Seq Library Preparation, and Ultra-Deep Next-Generation Sequencing (NGS)*

The targeted resequencing gene panel included coding exons and splice sites of 56 genes that emerged as drivers from a recent analysis of whole genome/exome data in more than 800 MM patients [1] (target region: 112 kb: *ACTG1, BCL7A, BHLHE41, BRAF, BTG1, CCND1, CDKN1B, CYLD, DIS3, DTX1, DUSP2, EGR1, FAM46C, FGFR3, FUBP1, HIST1H1B, HIST1H1D, HIST1H1E, HIST1H2BK, IGLL5, IRF1, IRF4, KLHL6, KMT2B, KRAS, LCE1D, LTB, MAX, NFKB2, NFKBIA, NRAS, PABPC1, PIM1, POT1, PRDM1, PRKD2, PTPN11, RASA2, RB1, RFTN1, RPL10, RPL5, RPRD1B, RPS3A, SAMHD1, SETD2, SP140, TBC1D29, TCL1A, TGDS, TP53, TRAF2, TRAF3, XBP1, ZNF462, ZNF292*). Tumor gDNA (median 400 ng) was sheared through enzymatic fragmentation before library construction to obtain 150–200-bp fragments. Targeted ultra-deep-next generation sequencing was performed by using the CAPP-seq approach, as described in Newman A.M. et al., Nat. Med. 2014 [2]. The NGS libraries were constructed using the KAPA Library Preparation Kit (Kapa Biosystems, Wilmington, MA, USA) and hybrid selection was performed with the custom SeqCap EZ Choice Library (Roche NimbleGen, Madison, WI, USA), allowing for enrichment by the capture of genomic regions of interest up to 7 Mb for human resequencing studies. Multiplexed libraries were sequenced using 200-bp paired-end runs on an Illumina MiSeq sequencer (Illumina, Hayward, CA, USA). Each run included 16 multiplexed samples, in order to allow >2000× coverage in >80% of the target region.

### *Bioinformatic Pipeline for Variant Calling*

We deduped FASTQ sequencing reads by utilizing FastUniq v1.1. The deduped FASTQ sequencing reads were locally aligned to the hg38 version of the human genome using BWA v.0.6.2, and sorted, indexed, and assembled into an mpileup file using SAMtools v.1. The aligned reads were processed with mpileup. Single nucleotide variations and indels were identified with a single-sample calling in tumor gDNA by using the germline function of VarScan2 *mpileup2cns* (a minimum Phred quality score of 30 was imposed). The variants revealed by VarScan2 were annotated by using SeattleSeq Annotation 151. Intronic variants mapping >2 bp before the start or after the end of coding exons, and synonymous variants, were filtered out. To account for the absence of a matched control, we filtered out variants reported by the database of genetic variation gnomAD (genome aggregation database) [3] as having an allele frequency greater than 1%, i.e., the allelic frequency typically taken as a threshold to define a variant as a polymorphism. To select variants with read counts significantly different from the expected baseline error, we used a Bonferroni-adjusted *p*-value of 8.905798e-8. To further filter out systemic sequencing errors, a database containing all background allele frequencies in all of the specimens analyzed was assembled. Based on the assumption that all background allele fractions follow a normal distribution, a Z-test was employed to test whether a given variant differs

significantly in its frequency from a typical DNA background at the same position in all of the other DNA samples, after adjusting for multiple comparisons by Bonferroni. Variants that did not pass this filter were not further considered, while retained variants were examined by means of several databases (FATHMM [4], MutationTaster2 [5], SIFT 4G [6], and PolyPhen-2 [7]) for the prediction of their functional impact/disease-causing potential, and by means of the COSMIC database to check whether they were cataloged in human cancer samples. In particular, we retained variants satisfying one of the three following criteria: reported in COSMIC as confirmed somatic; clustered (+/−3 codons) with mutations of the same class reported in COSMIC as confirmed somatic (defined as "oncogenic") [8]; and not classified as benign/tolerated by at least two of the four abovementioned function predictor databases. Variant allele frequencies for the resulting candidate mutations and the background error rate were visualized using Integrated Genome Viewer (IGV).

*ULP-WGS and Bioinformatic Analyses*

Ultra-low-pass whole genome sequencing (ULP-WGS) libraries were prepared using the Kapa HyperPlus kit (Roche, Madison, WI, USA) with SeqCap Library Adapters, starting with 400 ng of gDNA. Up to 24 libraries (2 μg DNA) were pooled and sequenced using 200 bp paired-end runs on a MiSeq (Illumina, Hayward, CA, USA), in order to obtain 2 million reads/sample, thus resulting in an average genome-wide fold coverage of 0.1×.

Raw FASTQ data were quality controlled with fastQC and trimmed to remove adapters with trimmomatic-0.38 (phred +33 and SLIDINGWINDOW:4:15 quality scores). The trimmed paired-end reads were aligned to the reference human genome (hg38) using a Burrows–Wheeler Aligner (BWA v.0.6.2). The duplicate reads were removed with the Picard tool, and unmapped reads were filtered out with samtools v.1.3.1. Then, the reads were post-processed following the Genome Analysis Toolkit (GATK) best practices 3.7, which combine the left alignment of small insertions and deletions, indel realignment, and base quality score recalibration. Finally, copy number alterations (CNAs) were predicted by using ichorCNA (https://github.com/broadinstitute/ichorCNA), with default parameters (window of 1000 kb), and a normal panel composed of 34 samples [9]. This computational method uses a probabilistic model, implemented as a hidden Markov model, to predict large-scale CNAs and estimate the tumor fraction of an ultra-low-pass whole genome sequencing sample. The software computes possible solutions in each sample, identified by a combination of the tumor fraction, ploidy, and subclonal portion. Then, the user manually chooses the most likely solution based on the tool output and the features of each patient.

*Chromosome 1p Copy Number Estimation Based on Targeted NGS Data*

By means of the PICARD tool (v2.22.2), we obtained, for each sample, the depth of coverage of four genes of the panel encoded on chromosome arm 1p (i.e., *FUBP1*, *RPL5*, *NRAS*, and *FAM46C*), and normalized it based on the total number of on-target mapped bases in that sample. We then estimated the copy number data of these loci in each of the four ichorCNA-deleted patients (060-BM, 100-BM, 136-BM, and 165-BM) by computing the logR ratio between their normalized depth of coverage and the mean normalized depth of coverage of ten samples without CNAs on chromosome arm 1p.

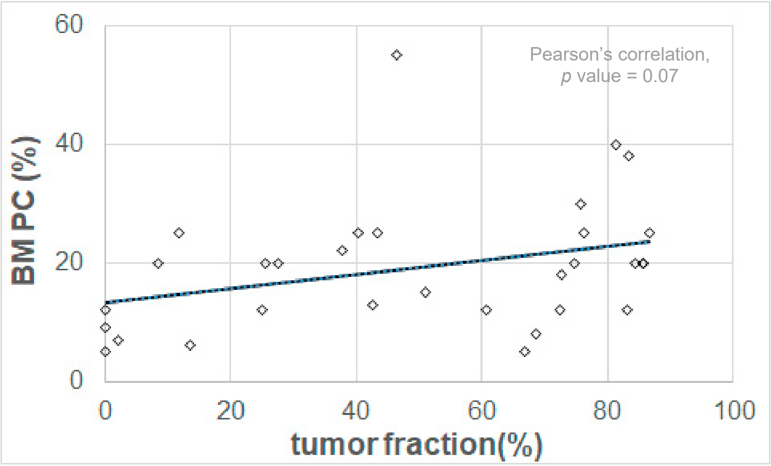


**Figure S1.** Tumor fraction and bone marrow (BM) plasma cell (PC) infiltration.

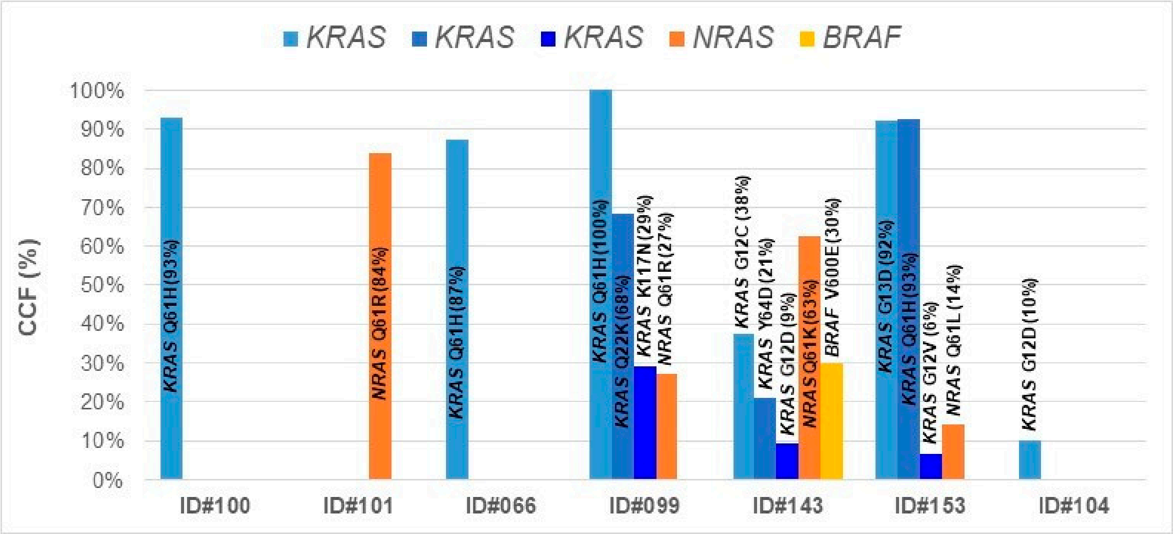


**Figure S2.** MAPK gene mutations. Bar chart visualizing the cancer cell fractions of mutations involving *BRAF*, *KRAS*, and *NRAS* genes. CCF, cancer cell fraction. CCF = min{1, f*[a*t + 2*(1−a)]/a*nCHR}, where f = variant allelic frequency, t = locus-specific copy number in tumor cells, a = tumor fraction, and nCHR = number of chromosomes bearing a mutation.

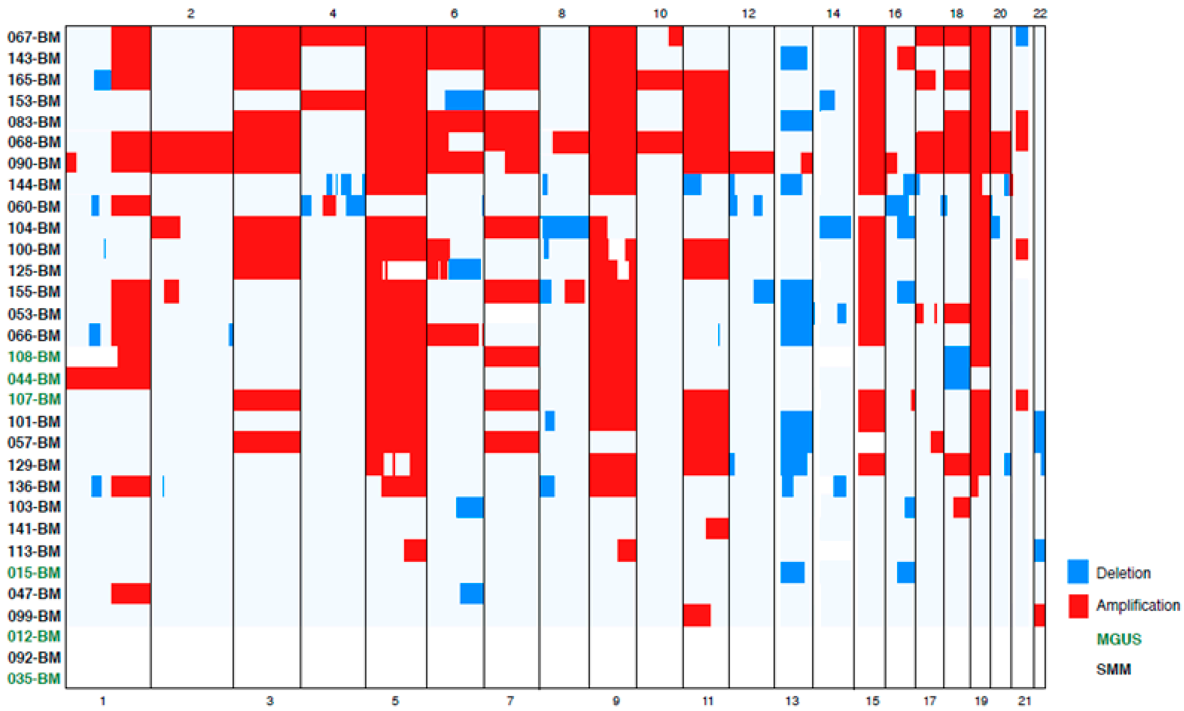


**Figure S3.** Heatmap of altered DNA regions in six MGUS and 25 SMM samples, as assessed by means of ichorCNA analysis of ultra-low-pass whole genome sequencing (ULP-WGS) data. Vertical axis: samples. Horizontal axis: chromosome localization. The samples were clustered according to their copy number (CN) values using the Euclidean distance and Ward linkage. Light blue: loss; white: normal CN; red: DNA gain/amplification (three or more copies). IDs corresponding to MGUS patients are printed in green.

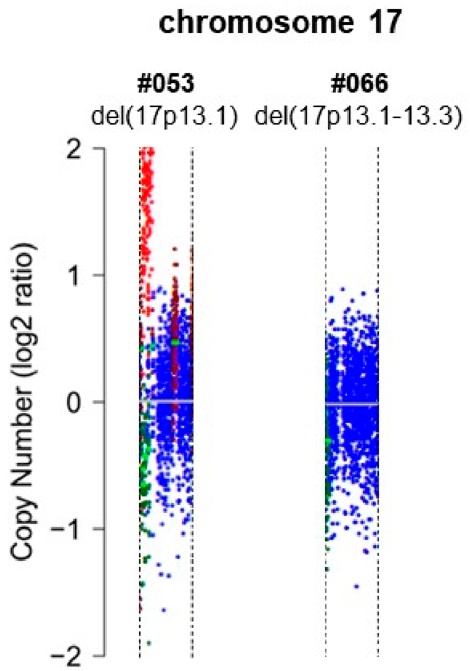


**Figure S4.** Detection of *TP53* gene deletion by an ichorCNA analysis of ULP-WGS data. Scatter plots portraying chromosome 17 copy ratios (y-axis) computed by ichorCNA in patient samples #053 and #066. X-axis coordinates represent nucleotide positions along chromosome 17. Red dots stand for amplification, brown ones for gain, green ones for loss, and blue ones for neutral copy number. The horizontal lines in light green indicate subclonal calls. This computation, obtained by setting genomic windows equal to 50 kb, allowed us to appreciate the occurrence of an interstitial deletion in each sample [del(17p13.1) in ID#053 and del(17p13.1-13.3 in ID#066], while analysis by default settings (genomic window = 1 Mb) failed to detect any chromosomal loss. The copy number pattern of chromosome 17 in patent ID#53 is suggestive of chromothripsis, inferred as described in Korbel J.O. and Campbell P.J., Cell 2013 [10].

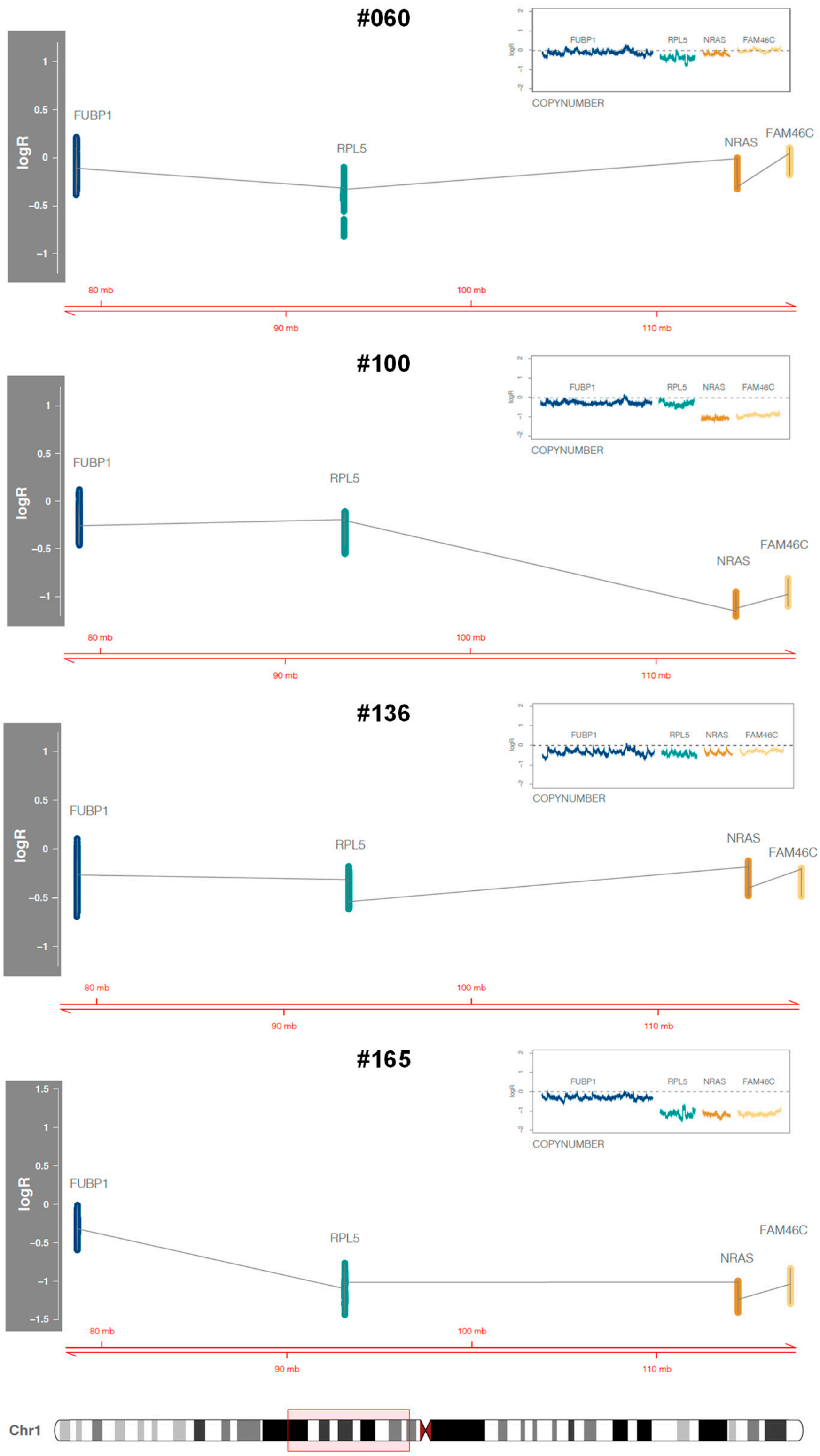


**Figure S5.** Detection of 1p-deletions based on targeted next-generation sequencing (NGS) data. Log ratio (logR) values (y-axis) of four genes of the panel encoded on chromosome arm 1p (i.e., *FUBP1*, *RPL5*, *NRAS*, and *FAM46C*) in four FISH-negative patients in whom ichorCNA revealed interstitial 1p deletions. In the main outline relative to each patient sample, selected loci (*FUBP1* in blue, *RPL5* in green, *NRAS* in brown, and *FAM46C* in yellow) are represented based on their genomic localization along the considered genomic region (red rectangle on the chromosome ideogram at the bottom). Each small top right panel shows the logR of the genes without considering the genomic distances, to underline the copy number variations.

**Table S1.** Non-synonymous mutations discovered by targeted gene mutation analysis.

| Absolute position* | Genes | RefSeq | Protein Change | Effect | AF gnomAD° | COSMICv91^ | FATHMM-MKL score§ | MutationTaster2 | SIFT 4G | PolyPhen-2 |
|---|---|---|---|---|---|---|---|---|---|---|
| NC_000012.12:g.122022142T>A | *BCL7A* | NM_020993.4 | p.ASP17GLU | missense | 0.00132% | somatic | 0.29877 | disease_causing | tolerated | possibly_damaging |
| NC_000012.12:g.122022143A>T | *BCL7A* | NM_020993.4 | p.ILE18PHE | missense | 0.00044% | oncogenic | 0.75446 | disease_causing | deleterious | possibly_damaging |
| NC_000012.12:g.122022182T>C | *BCL7A* | NM_020993.4 | p.TRP31ARG | missense | 0.00213% | oncogenic | 0.64011 | disease_causing | deleterious | benign |
| NC_000007.14:g.140753336A>T | *BRAF* | NM_004333.4 | p.VAL600GLU | missense | 0.00040% | somatic | 0.98542 | disease_causing | deleterious | probably_damaging |
| NC_000012.12:g.92144387A>C | *BTG1* | NM_001731.2 | p.ILE70SER | missense | na | oncogenic | 0.92314 | disease_causing | deleterious | probably_damaging |
| NC_000011.10:g.69641405T>A | *CCND1* | NM_053056.2 | p.MET31LYS | missense | na | na | 0.77439 | disease_causing | deleterious | possibly_damaging |
| NC_000011.10:g.69641420A>C | *CCND1* | NM_053056.2 | p.GLU36ALA | missense | na | oncogenic | 0.79804 | disease_causing | deleterious | benign |
| NC_000011.10:g.69641465A>G | *CCND1* | NM_053056.2 | p.GLU51GLY | missense | na | oncogenic | 0.76454 | disease_causing | deleterious | benign |
| NC_000016.10 :g.50779779C>G | *CYLD* | NM_015247.2 | p.SER418CYS | missense | na | na | 0.99324 | disease_causing | deleterious | probably_damaging |
| NC_000016.10 :g.50751643C>T | *CYLD* | NM_015247.2 | p.GLN182stop | nonsense | na | na | 0.98344 | disease_causing | na | na |
| NC_000013.11:g.72772200C>T | *DIS3* | NM_014953.3 | p.ASP488ASN | missense | na | somatic | 0.97693 | disease_causing | deleterious | probably_damaging |
| NC_000013.11:g.72761926C>G | *DIS3* | NM_014953.3 | p.ARG780THR | missense | na | somatic | 0.99869 | disease_causing | deleterious | probably_damaging |
| NC_000013.11:g.72763578A>T | *DIS3* | NM_014953.3 | p.MET667LYS | missense | na | somatic | 0.99363 | disease_causing | deleterious | probably_damaging |
| NC_000013.11:g.72772202T>A | *DIS3* | NM_014953.3 | p.ASP487VAL | missense | na | oncogenic | 0.99468 | disease_causing | deleterious | probably_damaging |
| NC_000002.12:g.96144879C>T | *DUSP2* | NM_004418.3 | p.GLY131ASP | missense | 0.00067% | somatic | 0.88976 | disease_causing | deleterious | probably_damaging |
| NC_000002.12:g.96144850C>G | *DUSP2* | NM_004418.3 | p.ASP141HIS | missense | 0.00070% | oncogenic | 0.87359 | disease_causing | deleterious | probably_damaging |
| NC_000002.12:g.96144883C>T | *DUSP2* | NM_004418.3 | c.389-1G>A | splicing | na | somatic | 0.648 | disease_causing | na | na |
| NC_000002.12:g.96145024G>C | *DUSP2* | NM_004418.3 | p.LEU111VAL | missense | na | oncogenic | 0.63921 | polymorphism | tolerated | possibly_damaging |
| NC_000004.12:g.1804404T>C | *FGFR3* | NM_001163213.1 | p.PHE386LEU | missense | 0.33570% | somatic | 0.82153 | polymorphism | tolerated | benign |
| NC_000004.12:g.1804840A>G | *FGFR3* | NM_001163213.1 | p.ASN430SER | missense | 0.00279% | oncogenic | 0.81635 | disease_causing | tolerated | benign |
| NC_000006.12:g.27867448C>G | *HIST1H1B* | NM_005322.2 | p.ALA28PRO | missense | 0.30550% | oncogenic | 0.00048 | polymorphism | tolerated | benign |
| NC_000006.12:g.26234377G>-CTT | *HIST1H1D* | NM_005320.2 | p.LYS185_ALA186delinsTHR | indel | 0.74160% | oncogenic | na | polymorphism | na | na |
| NC_000006.12:g.26234664G>C | *HIST1H1D* | NM_005320.2 | p.SER90ARG | missense | na | oncogenic | 0.04986 | disease_causing | tolerated | possibly_damaging |
| NC_000006.12:g.26156602A>G | *HIST1H1E* | NM_005321.2 | p.TYR71CYS | missense | 0.00040% | somatic | 0.41341 | disease_causing | deleterious | possibly_damaging |
| NC_000022.11:g.22888261T>G | *IGLL5* | NM_001178126.1 | c.206+2T>G | splicing | 0.00211% | somatic | 0.61489 | disease_causing | na | na |
| NC_000022.11:g.22888136T>A | *IGLL5* | NM_001178126.1 | p.LEU28GLN | missense | 0.00070% | oncogenic | 0.08646 | polymorphism | deleterious | benign |
| NC_000022.11:g.22888150A>T | *IGLL5* | NM_001178126.1 | p.MET33LEU | missense | 0.00068% | oncogenic | 0.03247 | polymorphism | tolerated | benign |
| NC_000005.10:g.132486310G>A | *IRF1* | NM_002198.2 | p.PRO203LEU | missense | 0.00419% | oncogenic | 0.98859 | disease_causing | deleterious | probably_damaging |
| NC_000003.12:g.183555469A>G | *KLHL6* | NM_130446.2 | p.LEU62PRO | missense | 0.00040% | oncogenic | 0.95926 | disease_causing | deleterious | probably_damaging |
| NC_000012.12:g.49032568C>T | *KMT2D* | NM_003482.3 | p.GLY4046GLU | missense | 0.00279% | oncogenic | 0.10145 | polymorphism | na | benign |
| NC_000012.12:g.49027073C>T | *KMT2D* | NM_003482.3 | p.ALA4965THR | missense | 0.00559% | oncogenic | 0.8537 | disease_causing | na | possibly_damaging |
| NC_000012.12:g.25227341T>G | *KRAS* | NM_004985.4 | p.GLN61HIS | missense | 0.00040% | somatic | 0.93196 | disease_causing | deleterious | benign |
| NC_000012.12:g.25245321G>T | *KRAS* | NM_004985.4 | p.GLN22LYS | missense | na | somatic | 0.99009 | disease_causing | deleterious | probably_damaging |
| NC_000012.12:g.25225713T>A | *KRAS* | NM_004985.4 | p.LYS117ASN | missense | 0.00040% | somatic | 0.91813 | disease_causing | deleterious | probably_damaging |
| NC_000012.12:g.25245350C>T | *KRAS* | NM_004985.4 | p.GLY12ASP | missense | 0.00140% | somatic | 0.97875 | disease_causing | deleterious | benign |
| NC_000012.12:g.25245351C>A | *KRAS* | NM_004985.4 | p.GLY12CYS | missense | na | somatic | 0.98367 | disease_causing | deleterious | probably_damaging |
| NC_000012.12:g.25227334A>C | *KRAS* | NM_004985.4 | p.TYR64ASP | missense | na | oncogenic | 0.9905 | disease_causing | deleterious | probably_damaging |
| NC_000012.12:g.25245347C>T | *KRAS* | NM_004985.4 | p.GLY13ASP | missense | 0.00140% | somatic | 0.97875 | disease_causing | deleterious | benign |
| NC_000012.12:g.25245350C>A | *KRAS* | NM_004985.4 | p.GLY12VAL | missense | na | somatic | 0.98367 | disease_causing | deleterious | probably_damaging |
| NC_000006.12:g.31581814C>G | *LTB* | NM_002341.1 | p.GLY70ARG | missense | 0.00082% | oncogenic | 0.76564 | disease_causing | tolerated | benign |
| NC_000010.11:g.102400429G>A | *NFKB2* | NM_001077494.3 | p.ARG579HIS | missense | 0.06631% | oncogenic | 0.42977 | polymorphism | tolerated | possibly_damaging |

| **Absolute position*** | **Genes** | **RefSeq** | **Protein Change** | **Effect** | **AF gnomAD°** | **COSMICv91^** | **FATHMM-MKL score§** | **MutationTaster2** | **SIFT 4G** | **PolyPhen-2** |
|---|---|---|---|---|---|---|---|---|---|---|
| NC_000001.11:g.114713908T>C | *NRAS* | NM_002524.4 | p.GLN61ARG | missense | na | somatic | 0.98635 | disease_causing | deleterious | benign |
| NC_000001.11:g.114713909G>T | *NRAS* | NM_002524.4 | p.GLN61LYS | missense | na | somatic | 0.99393 | disease_causing | deleterious | possibly_damaging |
| NC_000001.11:g.114713908T>A | *NRAS* | NM_002524.4 | p.GLN61LEU | missense | na | somatic | 0.98801 | disease_causing | deleterious | possibly_damaging |
| NC_000019.10:g.46704270A>G | *PRKD2* | NM_016457.4 | p.VAL263ALA | missense | na | oncogenic | 0.9815 | disease_causing | deleterious | probably_damaging |
| NC_000001.11:g.92833658C>G | *RPL5* | NM_000969.5 | p.GLN63GLU | missense | na | oncogenic | 0.96392 | disease_causing | deleterious | probably_damaging |
| NC_000020.11:g.36951501T>C | *SAMHD1* | NM_015474.3 | p.GLU48GLY | missense | 0.00070% | oncogenic | 0.80873 | disease_causing | deleterious | probably_damaging |
| NC_000003.12:g.47122861G>T | *SETD2* | NM_014159.6 | p.THR592LYS | missense | 0.08585% | oncogenic | 0.98241 | disease_causing | deleterious | benign |
| NC_000002.12:g.230311173T>A | *SP140* | NM_007237.4 | p.LEU768stop | nonsense | na | oncogenic | 0.55897 | disease_causing | na | na |
| NC_000002.12:g.230311523C>A | *SP140* | NM_007237.4 | p.HIS811GLN | missense | na | oncogenic | 0.00597 | polymorphism | tolerated | benign |
| NC_000002.12:g.230285833G>C | *SP140* | NM_007237.4 | c.1645+1C>G | splicing | na | oncogenic | 0.0337 | polymorphism | na | na |
| NC_000002.12:g.230292778G>A | *SP140* | NM_007237.4 | p.TRP653stop | nonsense | 0.00070% | oncogenic | 0.00913 | disease_causing | na | probably_damaging |
| NC_000014.9:g.95712362C>T | *TCL1A* | NM_021966.2 | p.ARG52HIS | missense | 0.03281% | oncogenic | 0.12322 | polymorphism | deleterious | probably_damaging |
| NC_000017.11:g.7673803G>A | *TP53* | NM_000546.5 | p.ARG273CYS | missense | 0.00120% | somatic | 0.98187 | disease_causing | deleterious | probably_damaging |
| NC_000017.11:g.7673740C>A | *TP53* | NM_000546.5 | p.GLU294stop | nonsense | na | somatic | 0.60338 | disease_causing | na | na |
| NC_000017.11:g.7673708A>-G | *TP53* | NM_000546.5 | p.THR304ILEfs*41 | frameshift | na | somatic | na | disease_causing | na | na |
| NC_000017.11:g.7674251A>G | *TP53* | NM_000546.5 | p.CYS238ARG | missense | na | somatic | 0.99392 | disease_causing | deleterious | probably_damaging |
| NC_000006.12:g.87259787C>-AG | *ZNF292* | NM_015021.2 | p.GLU2054LYSfs*14 | frameshift | na | somatic | na | disease_causing | na | na |
| NC_000009.12 :g.106939032G>A | *ZNF462* | NM_021224.4 | p.VAL2118ILE | missense | 0.00597% | somatic | 0.989 | disease_causing | tolerated | possibly_damaging |

*Absolute chromosome coordinates of each variant based on the GRCh38 version of the human genome assembly. AF = allelic frequency from the gnomAD browser that includes the gnomAD database v.3, gnomAD database v2.1.1, and ExAC database v1.0. ^Variants were classified as “somatic” if reported in COSMIC v91 as confirmed somatic, or “oncogenic” if clustered (+/−3 codons) with mutations of the same class reported in COSMIC as confirmed somatic [8]. §FATHMM is a method for predicting pathogenic point mutations: values above 0.5 are predicted to be deleterious, while those below 0.5 are predicted to be neutral or benign. na = not applicable.

**Table S2.** Patients' characteristics.

| ID | Gender | Age | Diagnosis | % BMPC | MC (g/dL) | MC type | FLC I/U ratio | Risk Group* | Follow-up (months) | TTP (months) |
|---|---|---|---|---|---|---|---|---|---|---|
| 107 | M | 44 | MGUS | 8 | *2.28* | κ | 3.74 | n.a. | | 25 |
| 12 | F | 60 | MGUS | 5 | 2.78 | κ | 1.42 | n.a. | 6 | |
| 15 | F | 62 | MGUS | 5 | 2.51 | κ | 1.68 | n.a. | 0 | |
| 108 | F | 79 | MGUS | 6 | 1.1 | κ | 1.2 | n.a. | 115 | |
| 44 | F | 69 | MGUS | 7 | n.d. | κ | 1.45 | n.a. | 7 | |
| 35 | M | 65 | MGUS | 9 | 0.5 | κ | 2.35 | n.a. | 113 | |
| 141 | M | 38 | SMM | 12 | 2.9 | κ | 18.76 | intermediate risk | 74 | |
| 53 | M | 76 | SMM | 20 | 2.5 | n.d. | 1.04 | intermediate risk | 84 | |
| 99 | M | 86 | SMM | 20 | 3.36 | κ | 9.3 | intermediate risk | 139 | |
| 153 | M | 80 | SMM | 20 | 3.9 | κ | 24.13 | high risk | 6 | |
| 136 | M | 58 | SMM | 25 | 2.5 | κ | 57.28 | high risk | | 51 |
| 90 | M | 76 | SMM | 20 | 1.6 | λ | 9.09 | low risk | | 53 |
| 144 | F | 77 | SMM | 55 | 2.4 | λ | n.d. | n.a. | 17 | |
| 155 | F | 70 | SMM | 18 | 1.63 | λ | 14.29 | low risk | 20 | |
| 125 | M | 73 | SMM | 20 | 4.5 | λ | 11.11 | intermediate risk | | 16 |
| 103 | F | 76 | SMM | 12 | 0.72 | λ | 7.69 | low risk | 98 | |
| 60 | M | 73 | SMM | 12 | 2.6 | κ | 1.02 | intermediate risk | | 51 |
| 165 | M | 70 | SMM | 13 | 2.3 | λ | 25.00 | high risk | 20 | |
| 83 | F | 45 | SMM | 22 | 3.8 | κ | 8.58 | high risk | 101 | |
| 100 | M | 71 | SMM | 38 | 1.7 | κ | 1.06 | intermediate risk | | 7 |
| 68 | M | 76 | SMM | 25 | 1.4 | κ | 39.14 | high risk | | 12 |
| 104 | M | 84 | SMM | 30 | 4.1 | κ | 11.59 | high risk | | 11 |
| 92 | M | 62 | SMM | 12 | 5.1 | κ/λ | n.d. | n.a. | 34 | |
| 67 | M | 48 | SMM | 20 | 1.3 | κ | 3.84 | low risk | 9 | |
| 129 | M | 93 | SMM | 12 | 0.9 | κ | 2.84 | low risk | 72 | |
| 47 | F | 50 | SMM | 25 | 1.9 | λ | 1.89 | intermediate risk | | 23 |
| 66 | F | 66 | SMM | 20 | n.d. | κ | 43.5 | n.a. | | 13 |
| 113 | M | 87 | SMM | 25 | *1.8* | κ | 13.96 | intermediate risk | 6 | |
| 143 | F | 57 | SMM | 25 | 3.3 | κ | 21.73 | high risk | 64 | |
| 101 | M | 80 | SMM | 40 | n.d. | κ | 1.49 | n.a. | | 7 |
| 57 | F | 68 | SMM | 15 | n.d. | κ | 2.95 | n.a. | 110 | |

Abbreviations: F, female; M, male; SMM, smoldering multiple myeloma; MGUS, monoclonal gammopathy of undetermined significance; BMPC, bone marrow plasma cell; MC, monoclonal component; FLC, free light-chain; n.a., not applicable; n.d., not determined. §Risk stratification based on the criteria published by Lakshman A. et al, BCJ 2018 [11].

## References


1. Maura, F.; Bolli, N.; Angelopoulos, N.; Dawson, K.J.; Leongamornlert, D.; Martincorena, I.; Mitchell, T.J.; Fullam, A.; Gonzalez, S.; Szalat, R.; et al. Genomic landscape and chronological reconstruction of driver events in multiple myeloma. *Nat. commun.* **2019**, *10*, 3835.
2. Newman, A.M.; Bratman, S.V.; To, J.; Wynne, J.F.; Eclov, N.C.; Modlin, L.A.; Liu, C.L.; Neal, J.W.; Wakelee, H.A.; Merritt, R.E.; et al. An ultrasensitive method for quantitating circulating tumor DNA with broad patient coverage. *Nat. med.* **2014**, *20*, 548–554.
3. Karczewski, K.J.; Francioli, L.C.; Tiao, G.; Cummings, B.B.; Alföldi, J.; Wang, Q.; Collins, R.L.; Laricchia, K.M.; Ganna, A.; Birnbaum, D.P.; et al. The mutational constraint spectrum quantified from variation in 141,456 humans. *bioRxiv* **2020**, 531210.
4. Shihab, H.A.; Rogers, M.F.; Gough, J.; Mort, M.; Cooper, D.N.; Day, I.N.; Gaunt, T.R.; Campbell, C. An integrative approach to predicting the functional effects of non-coding and coding sequence variation. *Bioinformatics* **2015**, *31*, 1536–1543.
5. Schwarz, J.M.; Cooper, D.N.; Schuelke, M.; Seelow, D. Mutationtaster2: Mutation prediction for the deep-sequencing age. *Nat. methods* **2014**, *11*, 361–362.
6. Vaser, R.; Adusumalli, S.; Leng, S.N.; Sikic, M.; Ng, P.C. Sift missense predictions for genomes. *Nat. protoc.* **2016**, *11*, 1–9.
7. Adzhubei, I.A.; Schmidt, S.; Peshkin, L.; Ramensky, V.E.; Gerasimova, A.; Bork, P.; Kondrashov, A.S.; Sunyaev, S.R. A method and server for predicting damaging missense mutations. *Nat. methods* **2010**, *7*, 248–249.
8. Bolli, N.; Biancon, G.; Moarii, M.; Gimondi, S.; Li, Y.; de Philippis, C.; Maura, F.; Sathiaseelan, V.; Tai, Y.T.; Mudie, L.; et al. Analysis of the genomic landscape of multiple myeloma highlights novel prognostic markers and disease subgroups. *Leukemia* **2018**, *32*, 2604–2616.
9. Adalsteinsson, V.A.; Ha, G.; Freeman, S.S.; Choudhury, A.D.; Stover, D.G.; Parsons, H.A.; Gydush, G.; Reed, S.C.; Rotem, D.; Rhoades, J.; et al. Scalable whole-exome sequencing of cell-free DNA reveals high concordance with metastatic tumors. *Nat. commun.* **2017**, *8*, 1324.
10. Korbel, J.O.; Campbell, P.J. Criteria for inference of chromothripsis in cancer genomes. *Cell* **2013**, *152*, 1226–1236.
11. Lakshman, A.; Rajkumar, S.V.; Buadi, F.K.; Binder, M.; Gertz, M.A.; Lacy, M.Q.; Dispenzieri, A.; Dingli, D.; Fonder, A.L.; Hayman, S.R.; et al. Risk stratification of smoldering multiple myeloma incorporating revised imwg diagnostic criteria. *Blood Cancer J* **2018**, *8*, 59.
